# Weak Molecular Interactions in Clathrin-Mediated Endocytosis

**DOI:** 10.3389/fmolb.2017.00072

**Published:** 2017-11-14

**Authors:** Sarah M. Smith, Michael Baker, Mary Halebian, Corinne J. Smith

**Affiliations:** School of Life Sciences, University of Warwick, Coventry, United Kingdom

**Keywords:** clathrin, endocytosis, adaptor-protein, structural biology, molecular interactions

## Abstract

Clathrin-mediated endocytosis is a process by which specific molecules are internalized from the cell periphery for delivery to early endosomes. The key stages in this step-wise process, from the starting point of cargo recognition, to the later stage of assembly of the clathrin coat, are dependent on weak interactions between a large network of proteins. This review discusses the structural and functional data that have improved our knowledge and understanding of the main weak molecular interactions implicated in clathrin-mediated endocytosis, with a particular focus on the two key proteins: AP2 and clathrin.

## Introduction

Biological processes are built on a complex interplay between proteins in the crowded, heterogeneous environment that exists within cells; and the functional protein interactions that are vital to these processes are often weak and transient. Insight into how such interactions are exploited in biological systems can help us understand how individual proteins contribute to functional networks and pathways.

One such pathway is clathrin-mediated endocytosis; a fundamental cellular process that serves to internalize cargo, that is—proteins or nutrients that need to be brought into the cell interior, and is implicated in numerous cellular functions including: nutrient uptake, membrane protein recycling, cell polarity, synaptic vesicle recycling and cell signaling. Defects in clathrin-mediated endocytosis have been linked to numerous pathological conditions such as Alzheimer's Disease, HIV/AIDS and hypercholesterolemia (Goldstein et al., 1985; McMahon and Boucrot, 2011; Zhang et al., 2011).

**Figure 1:**
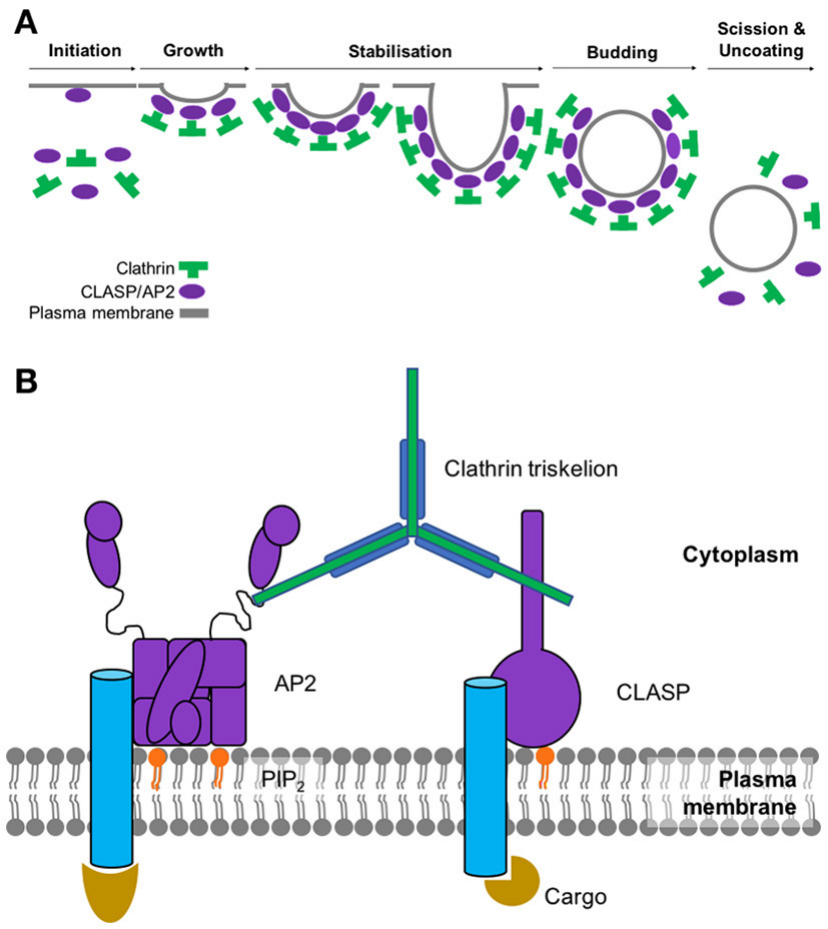
**(A)** Assembly and disassembly of clathrin-coated pit. Adaptor proteins associate at the membrane through interactions with phosphoinositides. AP2 and clathrin associated sorting proteins (CLASPs), such as AP180, interact with these membrane moieties, and once bound to the membrane, subsequently recruit clathrin triskelions to initiate lattice assembly. Recruitment of other adaptor proteins (e.g., Eps15, epsin, CALM/AP180) is required for stable lattice growth and vesicle closure. Dynamin, assisted by actin polymerization when the membrane is under tension, drives membrane scission and coated-vesicle release. Hsc70, recruited by the J-domain protein auxillin, mediates clathrin uncoating and release of a free vesicle, primed to fuse with a target membrane. **(B)** Key components involved in the initiation of clathrin-mediated endocytosis. Sites of active endocytosis are characterized by the accumulation of the key components: adaptor proteins, cargo, lipids and clathrin. Extracellular ligands (gold) are internalized by virtue of their signal-motif-bearing transmembrane receptor (blue) being recognized and bound by AP2 (or CLASP) (purple) at the intracellular side of the plasma membrane. CLASPs and AP2 bind to the PIP_2_ moieties of the inner membrane (orange). These proteins also serve to recruit individual clathrin triskelions (green) to the active endocytic site where their subsequent polymerization results in the formation of the clathrin coat.

The main stages of clathrin-mediated endocytosis can be subdivided into 6 main steps: initiation, growth, stabilization, vesicle budding, scission and uncoating (summarized in Figure 1A). As the name suggests, this type of endocytosis is characterized by its reliance on a protein called clathrin which interacts with a large network of adaptor proteins during the formation of a clathrin-coated vesicle and selection of cargo for internalization. Since clathrin cannot directly interact with the lipids or proteins of the plasma membrane (Maldonado-Báez and Wendland, 2006), adaptor proteins assist in the assembly of clathrin-coated vesicles by providing a link between clathrin and the membrane-bound cargo. The main adaptor protein that clathrin engages with at the plasma membrane is adaptor protein 2 (AP2). As well as binding to clathrin, AP2 also interacts with a significant number of binding partners which include receptors destined for internalization as well as other adaptor proteins that facilitate endocytosis (summarized in Figure 1B). AP2 is a member of a family of five heterotetrameric complexes. These complexes contain 4 types of subunit: two large (~100 kDa), one medium (~50 kDa), and one small (~17 kDa).

Key stages in clathrin-mediated endocytosis, such as receptor recruitment and assembly of the clathrin coat, appear to rely on weak interactions that are based on recognition of short peptide sequences. This review discusses how these weak molecular interactions are exploited by the crucial endocytic components: AP2 and clathrin.

## AP2 interactions—the molecular basis of receptor recruitment and clathrin coat formation

AP2 assists receptor internalization through several routes. It interacts directly with two types of internalization motifs (LL and (Y-X-X-Φ) (Φ = hydrophobic residue) found within the cytoplasmic domains of integral membrane protein receptors via its σ (LL) and μ2 (Y-X-X-Φ) subunits (Ohno et al., 1995; Owen and Evans, 1998; Owen et al., 2001; Collins et al., 2002; Kelly et al., 2008; Jackson et al., 2010). It is also associated with receptors indirectly by binding to other adaptors which are themselves (directly) associated with particular receptors, e.g., LDL receptor with autosomal recessive hypercholesterolemia (ARH) and G-protein coupled receptors (GPCRs) with arrestin.

The first receptor internalization motif to be identified was YXXΦ (Ohno et al., 1995). Surface plasmon resonance (SPR) experiments revealed that the YXXΦ motif binds to the μ2 subunit of AP2 with affinities between 10 and 70 μM (Boll et al., 1996; Rapoport et al., 1997). Owen and Evans (1998) gave a structural explanation for the affinity between the aforementioned tyrosine-based motifs and AP2. A 2.7 Å crystal structure of the signal binding domain of μ2 (residues 158–435) complexed with internalization signal peptides from EGFR (Sorkin et al., 1996) and TGN38 (Bos et al., 1993; Humphrey et al., 1993) revealed that hydrophobic pockets accommodate both the tyrosine and leucine residue of the sequence motif. Upon the target peptide binding, these pockets are positioned such that 3 additional H-bonds are made between the backbone of the peptide and the AP2, resulting in β-strand formation. A similar mechanism of increased binding affinity upon correct recognition of key side chains has also been shown in other cases (Lowe et al., 1997).

**Figure 2:**
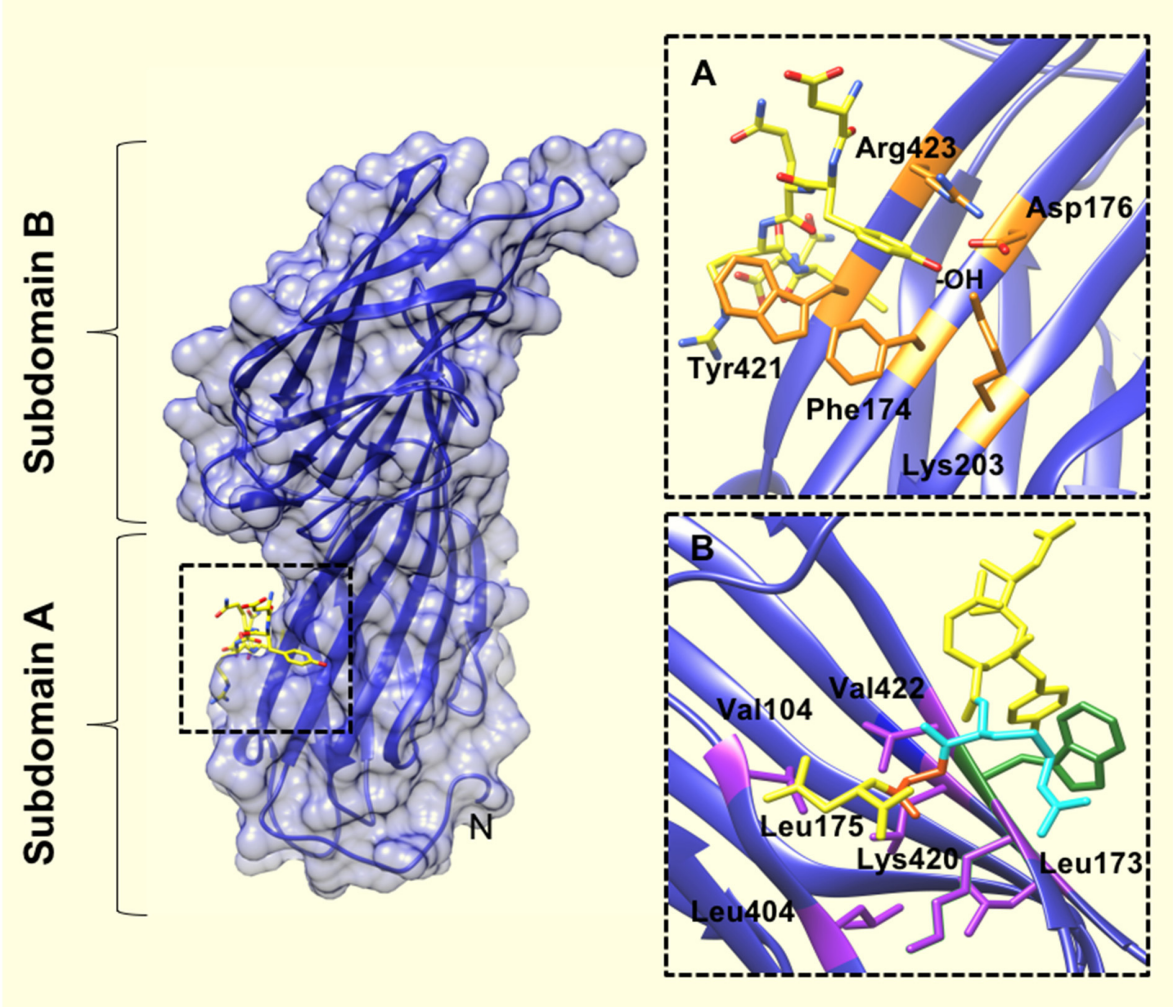
The YXXΦ peptide binding site of the μ2 subunit of AP2. **(A)** Upon binding of the TGN38 peptide (shown in yellow), the tyrosine residue of the YXXΦ motif binds in a hydrophobic pocket created by Phe^174^, Trp^421^, and Arg^423^. The tyrosine hydroxyl (indicated by, -OH) also engages in a network of hydrogen bonds with Asp^176^, Lys^203^, and Arg^423^. **(B)** The binding pocket for the bulky hydrophobic residue at position Y+3 of the YXXΦ motif (Leu in this instance), is lined with aliphatic side chains of residues (purple coloring): Leu^173^, Leu^175^, Val^401^, Leu^404^, Val^422^, and the aliphatic portion of Lys^420^. An Arg residue at Y+2 of the YXXΦ motif (cyan colored), packs against the Trp^421^ of u2 (green colored). PDB ID: 1BXX from, (Owen and Evans, 1998).

The tyrosine residue of the YXXΦ sequence motif forms significant interactions with the binding pocket. For example—there are hydrophobic interactions between the tyrosine ring and Trp^421^ and Phe^174^. In addition, the tyrosine hydroxyl engages in a network of hydrogen bonds with Asp^176^, Lys^203^, and Arg^423^. The bulky, hydrophobic residue (Φ) at position Y+3 of the internalization motif is also a major determinant of μ2 binding (Ohno et al., 1995; Boll et al., 1996), and binds in a cavity lined with aliphatic residues (Figure 2). Leu, Phe, Met or Ile residues at the Y+3 position could be accommodated in such cavity owing to the size and flexibility of side chains in the pocket.

Collins et al. (2002) obtained the structure of the AP2 core complexed with polyphosphatidylinositol headgroup mimic, inositolhexakisphosphate (IP6); which revealed two potential polyphosphatidylinositol binding sites: one on α and one on μ2. Interestingly, the YXXΦ binding motif (which localizes to the C-terminus of μ2) was occluded by part of the β2 trunk (Figure 2). This conformation of AP2 suggested to the authors a mechanism by which AP2 operates via an open or closed conformation in order to interact with motifs presented at the cell membrane.

This then raised the question of what the “open” conformation of AP2 might look like. Data showed that the distance between the end of a protein's transmembrane helix and a YXXΦ motif requires only seven amino acids in order to confer efficient internalization (Rohrer et al., 1996). In the “closed” AP2 core structure revealed by Collins et al, the YXXΦ binding site is ~65 Å from the membrane surface; therefore, AP2 must undergo a significant conformational change not only to expose the YXXΦ binding motif, but to also ensure that it is in close enough proximity to the transmembrane cargo.

**Figure 3:**
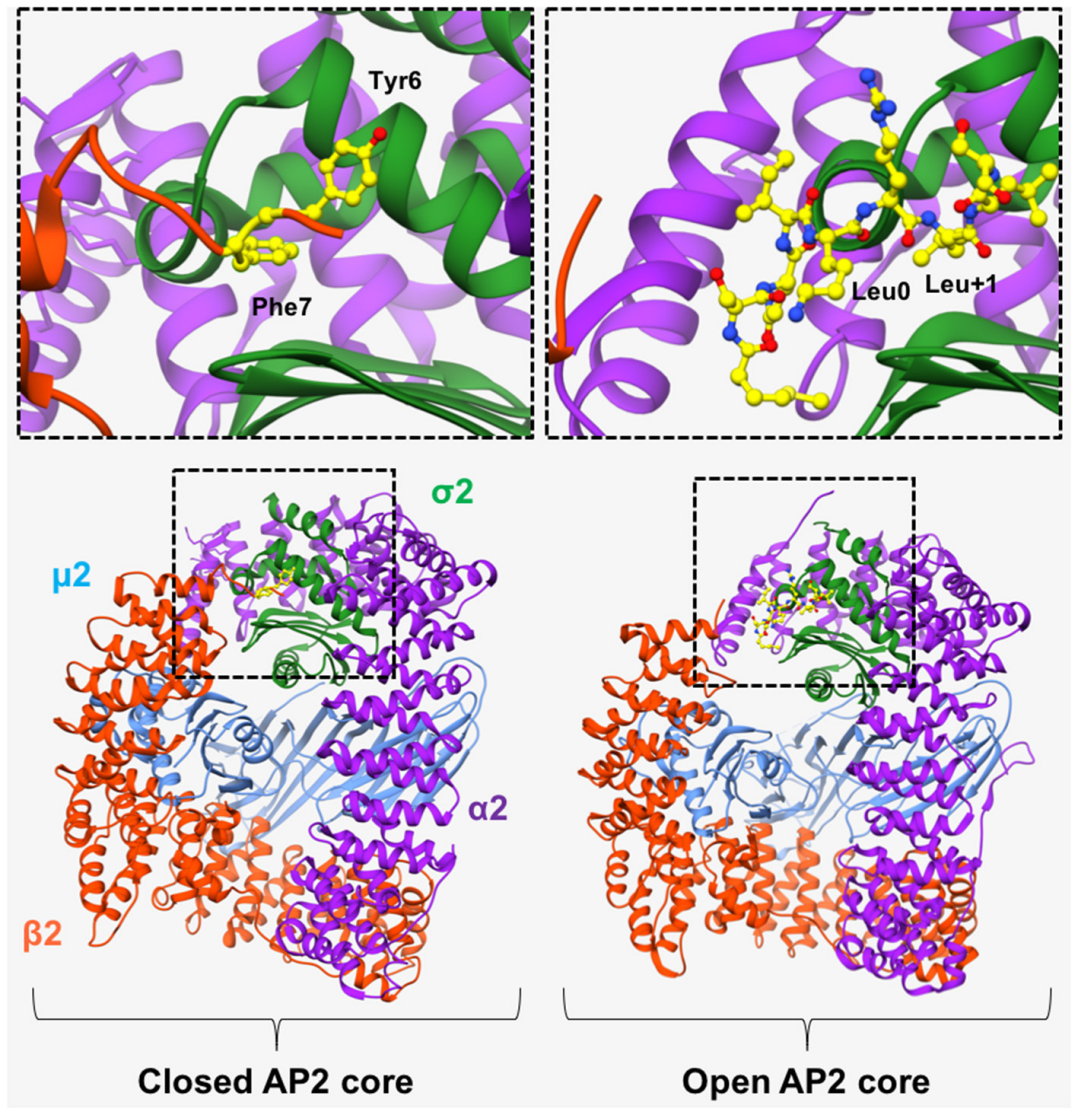
AP2 in “open” and “closed” conformation. (Left) In the basal, “closed” conformation of AP2, two N-terminal aromatic residues (Tyr^6^ and Phe^7^, shown in yellow in top left panel), obstruct the [DE]XXXL[LIM] binding site on σ2-subunit. Right) Binding of a CD4 peptide (shown in yellow in top right panel), causes the β2-subunit to move outwards, resulting in its N-terminus being expelled, and consequently exposing the hydrophobic binding pocket and allowing the LL-containing peptide to bind. However, the μ2 subunit remains closely associated with the β2 subunit and is therefore unable to bind YXXΦ motifs. α2 subunit—purple. μ2 subunit—blue. β2 subunit—orange. σ2 subunit green. PDB IDs: 2VGL from Collins et al. (2002) and, 2JKR from Kelly et al. (2008).

In 2008, Kelly et al. (2008) revealed the “open” conformation of AP2 upon crystallization of its core region bound to a peptide from CD4 (T-cell cell-surface antigen protein). Analysis of the crystal structure showed that the peptide bound to the core region in an extended conformation, with the LL moiety shown to bind 2 adjacent hydrophobic pockets on the σ2 subunit (Figure 3). SPR experiments showed that the CD4 LL-motif bound to WT AP2 with a Kd of 0.85 μM –considerably higher than the affinity previously shown for YXXΦ bound to the AP2 core. Comparison of this ligand-bound, “open” crystal structure with the previously published “closed” AP2 structure (Collins et al., 2002), showed that for the LL motif to bind, the N-terminus of β2 must be displaced from the surface of σ2, in order to expose the hydrophobic binding pocket (Figure 3).

Whilst the “unblocking” of the LL motif binding site was explained by minor conformational changes in AP2 core structure, (Kelly et al., 2008), the YXXΦ motif-binding site remained blocked. As mentioned above, the AP2 core must undergo substantial conformational changes to permit binding of membrane-embedded YXXΦ-containing cargo. To gain molecular insight into the large conformational change of AP2, Jackson et al. (2010) solved the crystal structure of a form of AP2 whereby both LL- and YXXΦ-motif binding sites are occupied. Driven partly by the phosphorylation of Thr156 on μ2 (Ricotta et al., 2002), and the electrostatic attraction of the highly positive electrostatic surface of the C-terminal region of this domain (C-μ2) to the negatively charged lipid head groups of the membrane, C-μ2 moves to the orthogonal face of the complex, resulting in the LL-motif, YXXΦ-motif and phosphatidyl inositol-4,5-bisphosphate (PtdIns4,5P_2_) –binding sites becoming coplanar on the surface of AP2 and therefore suitably positioned for contacting various motifs and/or signals at the plasma membrane. The adoption of an “open” AP2 conformation would therefore cause the β2 subunit to move out of the way and no longer occupy the motif binding sites.

Revelation of the “open” and “closed” conformations of the AP2 core structure was a significant milestone, providing mechanistic insight into how this adaptor protein is able to interact with internalization motifs found on the cytoplasmic tails of receptors. We have so far discussed how these interactions occur at the membrane, but in order for internalization to occur, the coated vesicle itself must form. This process of coat formation is driven by interactions between AP2 and its network of binding partners which bind to the appendage (or “ear”) domains of α2-adaptin and β2-adaptin. Here, weak interactions play a role as it was found that a number of adaptor proteins binding to the α2-appendage of AP2 did so via short linear motifs with weak binding affinities. An early crystal structure of this appendage revealed that the domain interface contains tightly packed and mostly hydrophobic residues. Hydrophobic surface potential analysis revealed a single candidate protein-binding sites that was centered around residue, W840 (Owen et al., 1999).

**Figure 4:**
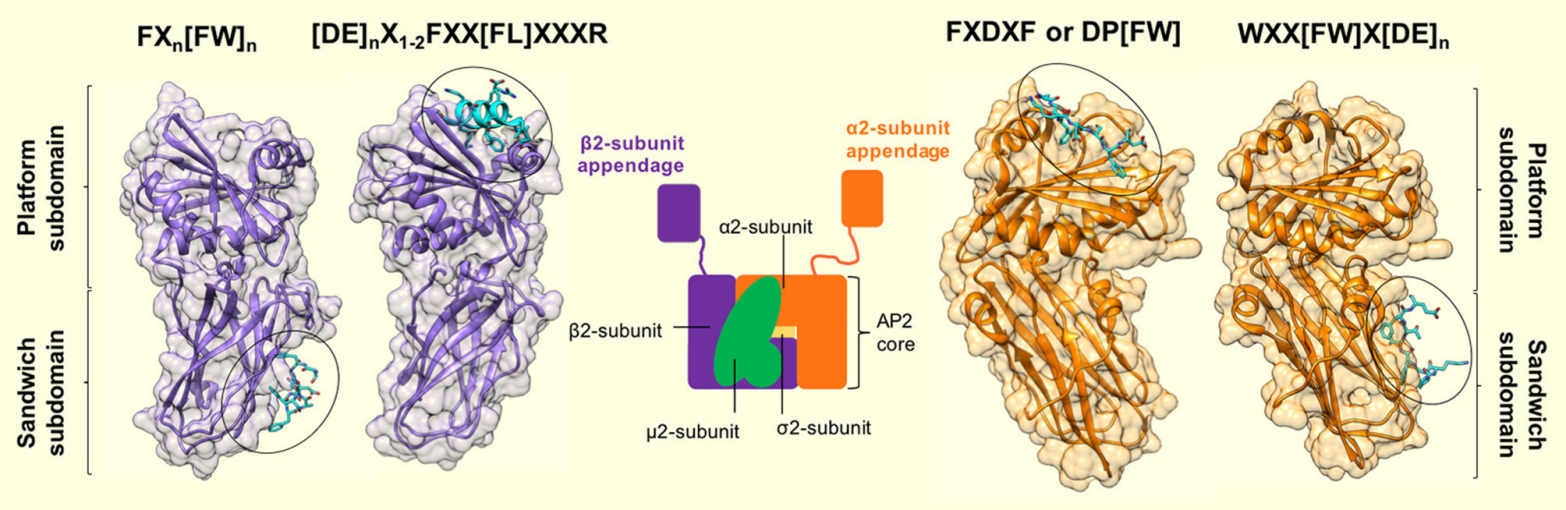
Motif binding to the appendage domains of AP2. The α2- and β2-subunit appendages of AP2 (orange and purple, respectively) share a similar bilobal structure each consisting of an N-terminal sandwich subdomain attached to a C-terminal platform subdomain. Each subdomain, of each appendage contains a distinct interaction surface for protein partner binding, which results in a single AP2 molecule possessing 4 separate contact sites. Specificity for each site is conferred by short, interaction motifs which are characterized by aromatic side chains. The sandwich subdomain of the α2-appendage binds to WXX[FW]X[DE]_n_-containing ligands (where X is any amino acid). [PDB code: 1W80, (Praefcke et al., 2004)]. The platform subdomain of the same appendage binds either FXDXF or DP[FW] motifs [PDB code: 1KY7, (Brett et al., 2002)]. The same subdomain of the β2-subunit binds to [DE]_n_X_1−2_FXX[FL]XXXR sequences that are presented in a α-helical conformation. Such motifs are present in β-arrestin, ARH and epsins (Edeling et al., 2006; Schmid et al., 2006) [PDB code: 2G30, (Edeling et al., 2006)]. Finally – the sandwich subdomain binds to a Phe-rich motif that is present in proteins, Eps15 and AP180 [PDB code: 2IV9, (Schmid et al., 2006)]. Peptide motif nomenclature as in Traub (2009).

Three linear motifs were found to bind the α2-appendage domain. These were DP[FW] (Owen et al., 1999; Brett et al., 2002), FXDXF (Collins et al., 2002), and WXX[FW]X[DE] (Ritter et al., 2003; Jha et al., 2004; Walther et al., 2004). Peptides containing these linear motifs were shown to bind the α2-appendage with relatively low affinities: 120 μM, 30–50 μM and 10 μM, respectively (Owen et al., 1999; Edeling et al., 2006). Furthermore, structural studies showed that peptides corresponding to these motifs bound to the α2-appendage in an extended conformation (Brett et al., 2002; Mishra et al., 2004; Praefcke et al., 2004; Ritter et al., 2004; Figure 4). Both the DPF/DPW and FXDXF motif bind to the α2-appendage through an overlapping site in the platform subdomain (Brett et al., 2002), whereas the WXX[FW]X[DE] motif was shown to interact with the sandwich subdomain (Praefcke et al., 2004; Ritter et al., 2004; Figure 4). This additional, distinct peptide binding site on the sandwich subdomain of the α2-appendage could permit multiple different motifs to bind the appendage, or, could allow multiple motifs of the same type to simultaneously bind, which would increase the avidity of the interaction (Walther et al., 2004).

The β2-appendage domain of AP2 was shown to possess a very similar bilobal structure to the α-appendage (Owen et al., 1999; Traub et al., 1999), with an N-terminal sandwich subdomain that is rigidly attached to a C-terminal platform subdomain (Owen et al., 2000). Also, as with the α-appendage, there was a single patch of highly hydrophobic surface potential on the β2-appendage platform subdomain that indicated a potential ligand interaction site. Charged residues adjacent to the hydrophobic pocket (such as R834, K842, E849, R879, E902, R904, and K917), could provide specificity for ligand-motif binding where the strength of the interaction was predominantly derived from hydrophobic interaction(s). For example, an abundance of positively charged arginine residues could confer electrostatic complementarity to a ligand rich in negatively charged amino acid side chains.

The β2-appendage domain binds a group of proteins that also bind the α2-appendage domain: AP180, epsin and eps15. Sequence analysis has shown that there is virtually no sequence homology between these proteins/ligands, except that they each contain multiple DPF/W sequences so the authors proposed that the DØF/W motifs are likely to mediate binding to the β2-appendage (Owen et al., 2000). Interestingly, these proteins bind AP2 appendage domains with differing affinities: the α2-appendage binds appreciable amounts of amphiphysin and has epsin as its high affinity ligand (Owen et al., 1999; Traub et al., 1999); whereas the highest affinity ligand for β2 appendage domain is eps15, with amphiphysin showing no significant signs of binding (Owen et al., 2000). This suggests that the context of the DPF/W motifs is also a factor in the interaction of these proteins with the AP2 appendage domains.

Owen et al. (2000) also showed that the β2 appendage together with its hinge region, is able to bind clathrin and also displace AP180, epsin and eps15 that is already bound to the domain. The binding region identified on the β2 appendage is larger and more open than the α2-appendage binding domain, which may explain its ability to preferentially bind clathrin. In light of these data, the author's proposed a model for CCV formation: in the cytosol, distant from regions of active endocytosis (Gaidarov et al., 1999; Roos and Kelly, 1999), AP2 appendage domains bind to DØF/W motif-containing accessory proteins such as AP180, epsin or eps15. In this region, the clathrin concentration is low (Goud et al., 1985; Wilde and Brodsky, 1996; Gaidarov et al., 1999) and would therefore be unable to compete with the aforementioned accessory proteins for AP2 binding. Conversely, at sites of active clathrin-mediated endocytosis, high clathrin concentrations would enable clathrin to compete effectively with DPF/W motif-containing accessory proteins for binding to the β2-appendage of AP2. Once bound, clathrin would be able to polymerize and consequently form a lattice, and recruit more clathrin.

A subset of the AP2 appendage binding accessory proteins are also able to bind cargo. These proteins, termed clathrin-associated sorting proteins (CLASPs), increase the catalog of endocytic cargo that can be recruited by AP2 beyond transmembrane proteins bearing cytoplasmic internalization motifs. Two key examples of the use of CLASPS are low density lipoproteins (LDL) and GPCRs, which are internalized by the CLASP proteins, ARH or Dab2 (Traub, 2005; Maurer and Cooper, 2006), and β-arrestin (Lefkowitz and Shenoy, 2005), respectively.

The binding of ARH to AP2 is highly selective for the β2-appendage (He et al., 2002; Laporte et al., 2002; Mishra et al., 2002). The 1.6 Å crystal structure of a β2-appendage in complex with an ARH-derived peptide (^252^DDGLDEAFSRLAQSRT) (Edeling et al., 2006), revealed a completely different mode of interaction in comparison to all other known appendage ligands. The ARH peptide adopted an α-helical conformation which bound a deep groove on the top of the β2-appendage (Figure 4). Analysis of the helix-binding region showed that Leu262 of the ARH peptide (termed [FL] pocket) was accommodated in a hydrophobic pocket of the β2 platform subdomain. Phe259 of the ARH peptide fitted into an adjacent, complementary hydrophobic pocket on the β2-appendage which the authors denote the “[F] pocket,” and the side chain of the residue Arg266 extends along a small channel on the surface of the β2 subdomain ([R] pocket) which forms hydrogen bonds with acidic residues Glu902 and Glu849. For these F, [FL] and [R] pocket interactions to occur, the α-helical motif must fit into its binding groove, therefore providing the specificity for binding. In agreement with this, a 2.5 Å crystal structure of the β2-appendage co-complexed with a Eps15 peptide and β-arrestin confirmed that the core motif for interaction with this AP2 appendage is: DxxFxxFxxxR, and exhibits an alpha helical conformation (Schmid et al., 2006).

Furthermore, β-arrestin, which has already been shown to bind the β2 appendage (Laporte et al., 2000; Kim and Benovic, 2002; Milano et al., 2002), displays significant sequence similarity with the β2-appendage binding motif of ARH at its C-terminus. Subsequent sequence analysis and mutagenesis of this C-terminal peptide region demonstrated the importance of the FXX[FL]XXXR motif in binding the platform subdomain of the β2-appendage (Edeling et al., 2006). Isothermal titration calorimetry (ITC) measurements confirmed that this region (^383^DDDIVFEDFARQRLKG) of β-arrestin binds the β2-appendage with a Kd of 2.6 μM; a value very similar to the Kd of ARH peptide of 2.4 μM (Mishra et al., 2005). Such affinity values are comparatively higher than those for a YXXΦ motif binding to the μ2 subunit of AP2 (Boll et al., 1996; Rapoport et al., 1997).

What's more, protein database searching revealed that mammalian epsins 1 and 2 also possess FXX[FL]XXXR motifs located in their unstructured region, which ITC experiments confirmed to bind the β2-appendage (Edeling et al., 2006). Further analysis showed that epsin, ARH and β-arrestin also contain acidic residues N-terminal to the proximal phenylalanine and thus the β2-appendage binding motif is more accurately described as: [DE]nX1–2FXX[FL]XXXR.

Taken together, these data reveal fundamental differences in the mode of interaction between the β2 platform domain and CLASPs compared to other appendage-ligand interactions. Instead of numerous, avidity-based interaction motif repeats, β-arrestin, ARH and epsin contain only a single [DE]nX1–2FXX[FL]XXXR motif, which adopts an α-helical conformation to bind the β2 appendage. Therefore, not only are there charge and hydrophobic components to the interaction between ligand and AP2, but extra specificity is conferred by the requirement that the CLASP motif folds into a helix with the interacting residues on one face of the helix.

In addition to high affinity interactions between AP2 appendages and accessory proteins bearing a single appendage binding motif, α2- and β2-appendages also engage in high avidity interactions. SPR experiments between immobilized α2- or β2-appendages and the motif domain of Eps15, showed very tight interactions such that an off-rate could not be measured (Schmid et al., 2006). It was assumed that these tight interactions were due to the presence of multiple appendage interaction sites in a single protein domain of Eps15. Therefore, if appendages are linked/bound to the same surface there is a high avidity for ligand interaction that is much stronger than the sum of individual affinities (Praefcke et al., 2004). So in the context of clathrin-mediated endocytosis, such an environment would be akin to “assembly-zones,” where AP2 is clustered at the membrane, presenting multiple appendages that are available for accessory protein binding. Proteins with multiple appendage interaction sites will not only aid adaptor clustering, but the presentation of many juxta-positioned motifs leads to an increased affinity for the adaptor appendage. Therefore, individual weak affinity interactions between AP2 and its ligands can make significant contributions to protein-protein interactions, providing there are multiple copies.

Schmid et al. (2006) proposed that clathrin coated pit (CCP) formation proceeds as a result of high avidity interactions of accessory proteins being replaced by the weak interactions of the clathrin coat with adaptors; meaning that initially low affinity (and therefore readily reversible) interactions between cargo and adaptors, between adaptors and accessory proteins, and between accessory proteins and clathrin, are used to build the network.

## Clathrin-adaptor interactions mediated by short peptide motifs

**Figure 5:**
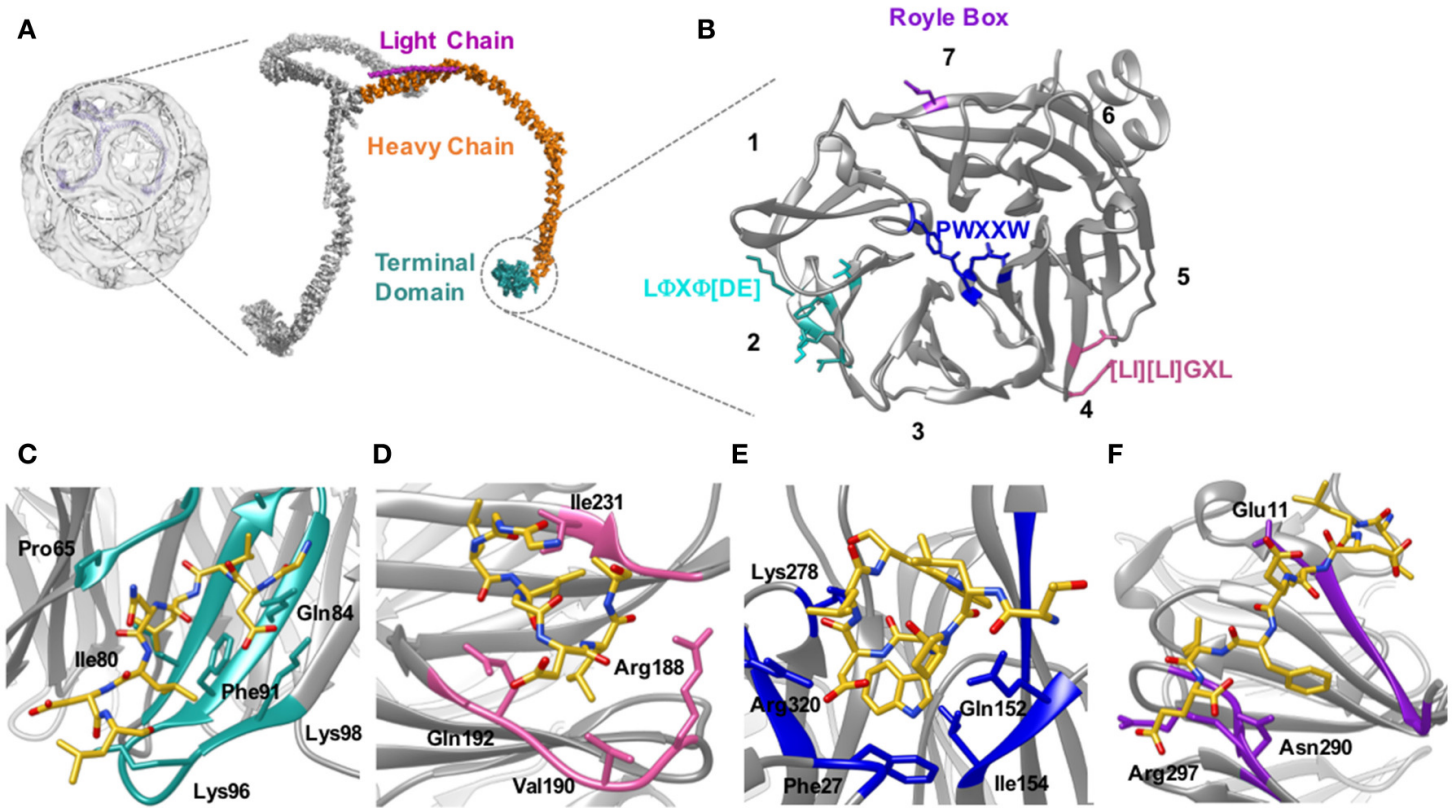
Location of the clathrin heavy chain N-terminal domain (TD) and the location of the adaptor binding sites. **(A)** Clathrin forms a polymerized lattice structure around the growing vesicle in concert with adaptor proteins. The functional monomer, the triskelion, is formed of a trimer of heavy chains (~190 kDa, orange) with a smaller light chain (~25 kDa Pink) located along the top edge near the dimerization domain. The TD (cyan) is the primary binding location for adaptor proteins, and is located on the inside of the cage closest to the plasma membrane. **(B)** The TD is a 7-bladed β propellar that has 4 known sites for binding adaptor proteins. Site 1 between blades 1 and 2 is known as the clathrin box site (LΦXΦ[DE]), (turquoise); Site 2 situated in the center of the propellar is known as the W-box (PWXXW), (blue); Site 3 is the Arrestin-box ([LI][LI]GXL), (Pink); and Site 4 is the Royle Box which as yet has no defined interaction sequence, (purple). The numbers indicate the blade number. Peptides or protein bound with the 4 sites are indicated in the following four panels: **(C)** Clathrin box site of β2 adaptin (CGDLLNLDLG) bound to site 1; **(D)** βarrestin1L peptide (ALDGLLGG) bound to site 3; **(E)** amphiphysin peptide (TLPWDLWTT) bound to site 2; **(F)** an amphiphysin peptide bound to site 4. Structures are derived from PDB codes: 3IYV **(A)** (Fotin et al., 2004), 5M5R **(B,C)** (Muenzner et al., 2017), 3GD1 **(D)** (Kang et al., 2009), 1UTC **(E)** (Miele et al., 2004), 5M5T **(F)** (Muenzner et al., 2017).

A pivotal step upon the recruitment of clathrin to sites of endocytosis is the interaction between individual clathrin triskelia and an array of accessory proteins that assist in the formation of a clathrin-coated pit. Clathrin has more than 20 binding partners and interacts with most of these via a 7-bladed beta-propeller domain at its N-terminus (Figures 5A,B). Interactions between the clathrin N-terminal domain (TD) and peptides corresponding to multiple binding motifs are in the micromolar range. Thus, weak interactions also feature in the role of clathrin as well as AP2 in endocytosis.

The mode of binding of adaptor proteins for clathrin was investigated by Dell'Angelica et al. (1998) using a combination of GST pull-down assays and mutagenesis. Through this they identified a segment of residues (SLLDLDDFN817–825) in the β3-appendage of AP-3 that contributed to binding to the clathrin TD. Subsequent sequence analysis also identified similar amino acid residues in other adaptor proteins that have been shown to mediate interaction(s) with clathrin; namely—amphiphysin II (Ramjaun and McPherson, 1998), segments of arrestin3 (Krupnick et al., 1997) and in the clathrin-binding region of β1 and β2 (Shih et al., 1995). Alignment of these sequences enabled the definition of a motif for clathrin-binding, comprised of acidic and bulky hydrophobic residues, L(L, I)(D, E, N)(L, F)(D, E), termed the “clathrin box” motif (Figures 5B,C). Although they vary between proteins, clathrin-box motifs are highly-conserved and are found in many proteins known to interact with the TD, for example—AP1, epsin, AP180 and amphiphysin (Shih et al., 1995; Drake et al., 2000; Kirchhausen, 2000).

A number of X-ray structures of the clathrin TD provided both its tertiary structure and the binding location of peptides from several different adaptor proteins, revealing 3 independent binding sites for clathrin binding partners on this relatively small (40 kDa) (ter Haar et al., 2000) domain.

Almost two decades ago, ter Haar et al. (1998) showed that the clathrin TD comprised a 7-bladed beta-propeller structure linked to a series of short alpha helices which formed the start of the clathrin “leg” region. ter Haar et al. (2000) then determined structures of complexes of clathrin TD with peptides derived from adaptors β-arrestin 2, and the β-subunit of AP3. The residues contacting the TD in both structures were consistent with the five-residue clathrin box motif identified previously: LΦXΦ[DE] (where x denotes any amino acid, Φ denotes a bulky hydrophobic residue and [DE] is a glutamate or aspartate). Both structures revealed very similar peptide interactions with each peptide binding in an extended conformation in a groove between blades 1 and 2 of the propeller structure (Figures 5B,C). The sharing of the same binding site by both peptides was surprising given previous evidence suggesting that β-arrestin 2 and AP2 could bind at different sites on the TD (Goodman et al., 1997).

The situation became more complex when a 2.3 Å crystal structure of clathrin TD bound to a peptide of amphiphysin 1 revealed a second TD-binding motif, PWXXW (termed “the W-box”), to bind at a site remote from the “clathrin-box” binding site (Miele et al., 2004; Figure 5B). The presence of a second motif-binding site had previously been suggested by biochemical data which indicated that the binding sequence, PWDLW, could bind to the TD without competing with the canonical LΦXΦ[DE] clathrin-box motif (Ramjaun and McPherson, 1998; Slepnev et al., 2000; Drake and Traub, 2001).

Unlike the clathrin-box motif (which adopts an extended conformation when bound to the TD), the bound W-box was compact and helical, buried in a solvent-exposed cavity of complementary shape on the membrane-proximal “top” surface of the TD; a location spatially distinct from where the clathrin-box peptides bind (Miele et al., 2004; Figure 5E). Affinity measurements showed that the W-box peptide binds the TD with a similar affinity (Kd of 28 μM) to that of the clathrin-box peptide (Kd of 22 μM).

Finally, a third, spatially distinct adaptor binding site was identified by Kang et al. (2009), who showed that an extended surface loop of the arrestin 2 long isoform occupied a site between blades 4 and 5 of the TD, which binds peptides with motif [LI][LI]GxL– termed the “arrestin-box” (Figures 5B,D).

The above crystal structures revealed the location of adaptor protein binding sites to the TD; however, the role of these interactions in coat assembly has been difficult to define. The structures obtained so far are of peptides corresponding to clathrin-binding motifs co-crystallized with the clathrin TD. It would be interesting to know how TD binding to the full-length clathrin-binding domains of these proteins compares but this will be hard to achieve by crystallography (ter Haar et al., 2000; Miele et al., 2004) owing to the unstructured nature of these clathrin binding regions. AP180, implicated in clathrin assembly, is one such example. It has a 33 kDa N-terminal ANTH domain, which is involved in membrane binding; and a largely unstructured 58 kDa C-terminal region that is responsible for clathrin-binding and assembly. More specifically—the C-terminal region region binds to the TD of the clathrin heavy chain (Morgan et al., 2000), and self-homology analyses of this region showed that it contains 12 repeats, each ~23 aa in length, and containing a single DLL/DLF sequence per repeat. The large number of clathrin binding motifs along the length of the AP180 sequence suggests that organization of AP180 binding to clathrin must go beyond a straight forward 1:1 interaction between a single DLL motif and the clathrin TD.

## More complex interactions—multiple DLL motifs

The potential for complex binding interactions between clathrin binding motifs and clathrin TD led Zhuo et al. (2010) to further investigate the binding of DLL and DLF motifs in AP180 to clathrin TD. Zhuo et al. (2010) demonstrated that the DLL and DLF sequences within the clathrin binding site are critical for clathrin binding, and bind clathrin TD relatively weakly, with Kd values in the ~2 × 10^−4^ M range. The weak binding of these sites to the clathrin TD and the observation that chemical exchange kinetics are in the intermediate to fast-exchange regimen (Schlosshauer and Baker, 2004) indicate that both association and dissociation rates for these interactions are rapid, with dissociation rates in the range of 2 × 10^3^ and 4 × 10^3^ s^−1^, and association rates in the range of 1 × 10^7^ to 2 × 10^7^ M^−1^ s^−1^.

In light of these data, the authors were able to expand on previous models for how AP180 mediates clathrin coat assembly. AP180 binds to the membrane via its ANTH domain, resulting in its unstructured, flexible C-terminal region being exposed to the cytoplasm and available for binding any clathrin molecules encountered. Although the clathrin binding site of AP180 binds clathrin weakly with rapid dissociation rates, the likelihood of clathrin diffusing away is minimized given that each AP180 molecule contains up to 12 clathrin binding sites; therefore, if a clathrin molecule unsuccessfully binds one site, it is possible that it can interact with the many other clathrin binding sites. What's more, the rapid dissociation rates mean that each triskelion is able to move and reorient itself, enabling interactions with other triskelia to be established. Those clathrin-clathrin interactions that occur will determine both the geometry and stability of the clathrin lattice. In this way, weak binding by multiple clathrin triskelia to binding sites dispersed throughout the AP180 sequence allows efficient recruitment of clathrin to endocytic sites and dynamic assembly of the clathrin lattice. This example of weak, multi-valent binding in combination with intrinsic disorder of a protein binding partner is able to create a highly dynamic mode of protein-protein interaction.

## Complexity of binding to multiple clathrin terminal domain sites

The crystal structures of ter Haar et al. (2000), Miele et al. (2004), and Kang et al. (2009) suggested that clathrin-box motif, W-box motif and arrestin splice loop 5 peptides bind uniquely to individual sites on the TD, respectively, giving a total of 3 binding sites. However, data published since then has suggested that such a situation is likely to be an oversimplification. For example, yeast epsin (Ent2p) was shown to bind a TD where clathrin-box and W-box binding sites were mutated. Additionally, deletion of the Ent2p C-terminal clathrin-box sequence eliminated Ent2p binding to the TD (Collette et al., 2009). Together, these data indicate that clathrin-box sequences are able to bind the TD at site(s) distinct from site 1 or 2. In fact, a fourth adaptor binding site was identified by Willox and Royle (2012). This study found that mutating all 3 binding sites did not block clathrin/AP2 mediated endocytosis in human cell lines whereas deleting the TD inhibited endocytosis. Using an *in silico* approach they were able to identify a conserved patch located ~120° relative to the other binding sites. This site encompasses the end of strand d of blade 7 and the helical segment in the loop connecting blades 7 and 1 (Figures 5B,F). Mutating E11 to K on this 4th site, in combination with mutations to the other 3 sites, resulted in the same phenotype as shown by deletion of the TD. The identity of the motif conferring binding to this site remains undefined.

A deeper understanding of adaptor binding to the four, distinct adaptor-binding sites on the clathrin TD must account for observations that three out of four of the aforementioned binding sites can be mutationally eliminated without causing loss of CME (Willox and Royle, 2012).

In an effort to gain insight into the ambiguities regarding adaptor/accessory protein binding to clathrin TD, Zhuo et al. (2015) adopted a solution-based NMR approach to study the interaction of clathrin TD with clathrin-box peptides derived from AP2 adaptor protein and the accessory protein, AP180. Results showed that these peptides simultaneously bound the clathrin-box site, the W-box site *and* the β-arrestin splice loop site of a single TD with a similar, low affinity (Kd values in the range of 800–900 μM). The high promiscuity and stoichiometry of binding of peptide to the TD could be a reflection of the functional redundancy of these sites, and could also be important for the dynamic reorganization of the clathrin TD during endocytosis.

In agreement with biochemical data that showed clathrin only precipitated in GST-binding assays upon immobilization of a high density of clathrin-box peptides to a GST-resin (Drake et al., 2000; Drake and Traub, 2001), the weak molecular interactions between clathrin-box peptides and the clathrin TD (Zhuo et al., 2015) means that multiple interactions are required for a stable association of adaptor/accessory protein with clathrin. Furthermore, these data also suggest that each TD can bind up to 3 such peptides, which not only increases the potential avidity of peptide-TD interaction, but also offers an explanation as to why individual binding sites in the TD can be mutationally eradicated without significantly compromising CME (Lemmon and Traub, 2012). Also, it has been proposed that weak molecular interactions between TD and peptides would facilitate the dynamic reorganization of clathrin during lattice assembly (Zhuo et al., 2010). The temporal regulation of this event and the fact that it involves transfer of clathrin between different adaptor and accessory proteins during the process of internalizing cargo (Drake et al., 2000; McMahon and Boucrot, 2011), enables the development of our understanding of clathrin-mediated endocytosis. The authors (Zhuo et al., 2015) propose that the differing affinities and number of clathrin binding sequences in an adaptor/accessory protein could be an important factor in aiding clathrin transfer: tighter binding, or more clathrin binding sequences could displace a protein that has weaker or fewer clathrin binding elements.

Muenzner et al. (2017) endeavored to investigate the suggested potential degeneracy of clathrin binding, (Willox and Royle, 2012; Zhuo et al., 2015) by resolving high resolution structures of clathrin TD complexed with cellular and viral peptide motifs. In contrast to previous crystallographic structures (ter Haar et al., 2000; Miele et al., 2004), where the co-complexed peptide was shown only to bind a single site of the clathrin TD, the structures resolved by Muenzner and colleagues demonstrated that 2 distinct sequence motifs (arrestin-box and the clathrin-box), can bind the arrestin box binding site of clathrin TD.

Furthermore, the authors also note that the sequences capable of binding the Royle box are somewhat variable (amphiphysin I clathrin-binding motif peptide: ETLLDLDFLE and hepatitis D virus large antigen peptides: SDILFPADS and SPRLPLLES), preventing the unambiguous identification of a consensus binding sequence. Thus, they suggest that the model of “1 consensus motif binds a single peptide-binding site on the clathrin TD” may require revision since binding could rely on the peptide's structural environment upon contacting the TD (Muenzner et al., 2017).

The fact that a clathrin TD is capable of simultaneously binding multiple adaptors emphasises the dynamic nature of clathrin-adaptor interactions. The authors (Muenzner et al., 2017) go on to discuss that differences in the affinity of protein-protein interactions come as a result of differing rates of dissociation (Pollard, 2010). Weak molecular interactions, such as those between clathrin and its adaptor proteins (Shih et al., 1995; Zhuo et al., 2015), are on the order of approximately 1 per second (Pollard, 2010). Given that the timeframe of complete clathrin-coated pit formation and disassembly is ~90 s (Loerke et al., 2009), we would anticipate that adaptors undergo rapid cycles of binding and dissociation from clathrin, which would enable the recruitment of many different adaptor proteins to a given clathrin TD. Also, the promiscuity of clathrin motif binding would permit a single adaptor protein that contains multiple clathrin interaction motifs (e.g., AP180 Zhuo et al., 2010), to simultaneously bind multiple sites on clathrin TD, consequently increasing the affinity of the interaction.

## Conclusion

This review of the structural and functional experiments that investigated the binding between cargo, adaptor and accessory proteins, as well as clathrin, has demonstrated the different ways weak molecular interactions are exploited in clathrin-mediated endocytosis.

AP2 is a key regulatory factor in clathrin-mediated endocytosis, and its activation commences upon recruitment and subsequent low-affinity interactions with PtdIns4,5P2 of the plasma membrane (Höning et al., 2005). Phosphorylation of Thr156 of the μ2 domain causes AP2 to adopt an “open” conformation allowing it to interact with the large network of other accessory proteins and clathrin.

The evolution of AP2 to adopt “open” and “closed” conformations allows this protein to spatially and temporally control different stages of cargo internalization, from the point of cargo recognition and sorting, to downstream clathrin-coated pit formation.

**Figure 6:**
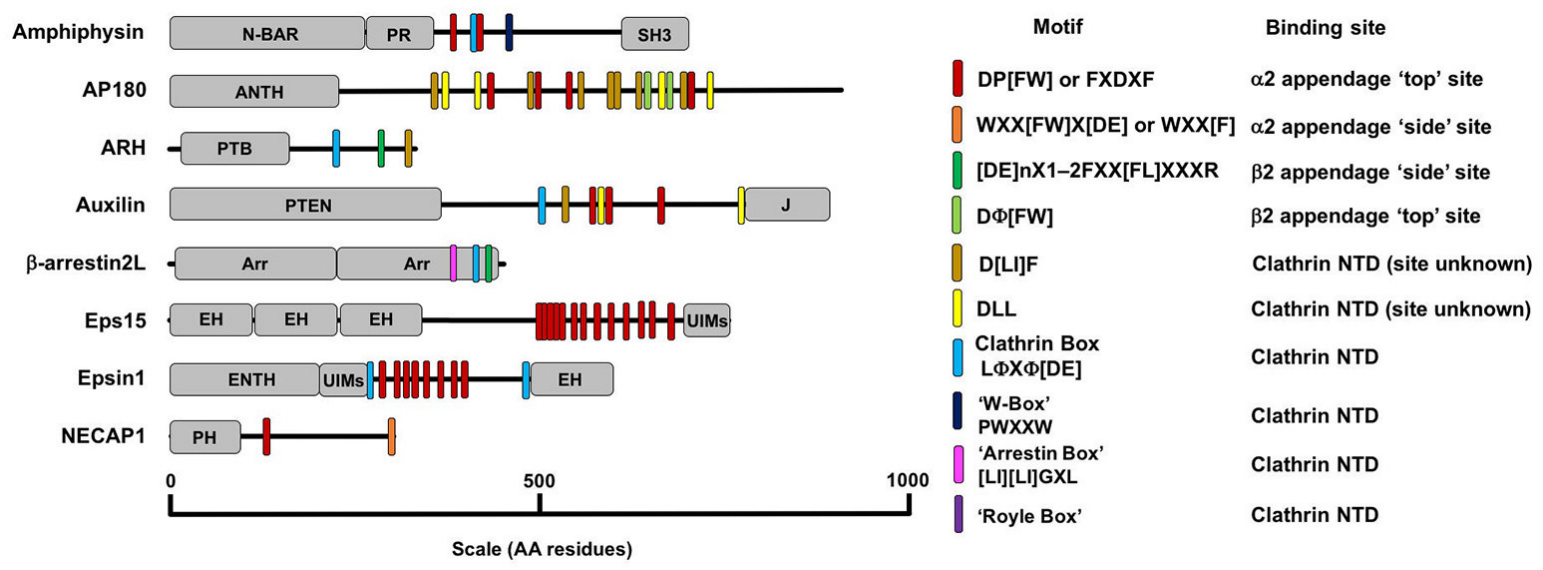
A diagram detailing the AP2 and clathrin binding motifs present in a number of adaptor proteins with diverse structure and function. Motifs are listed in the key on the right along with their binding location on AP2 or clathrin. The other domains detailed in the figure are as follows: ANTH, AP180 N-Terminal Homology Domain, Arr, Arrestin Domain; CC, coiled-coil domain; EH, Epsin Hand; ENTH, Epsin N-Terminal Homology Domain; J, J-domain; SH3, SRC Homology domain 3; PH, Plextrin Homology domain; PR, Proline Rich domain; PTB, Phosphotyrosine Binding domain; N-BAR, N-terminal Bin/Amphipysin/Rvs domain; UIM, Ubiquitin Interacting Motif.

Together, the AP2 core and its appendage domains bind their respective ligands via motifs (summarized in Figure 6), which engage in weak molecular interactions. This network of low-affinity protein interactions provides not only high avidity and specificity, but also reversibility of protein interactions that allows for rapid exchange of binding partners, accounting for the dynamic nature of CME *in vivo* (Avinoam et al., 2015).

Whilst low-affinity interactions are common throughout clathrin-mediated endocytosis, AP2 is also able to engage in high affinity interactions with some accessory proteins. These interactions are conferred by the requirement that a ligand motif binds in an α-helical conformation, as opposed to an extended conformation that is more commonly used. These two different modes of interaction between AP2 appendages, along with the multiple variants in binding motifs, explain how AP2 is able to act as a central hub for interactions between adaptors and clathrin. However, a greater understanding of how these motifs interact with and compete with each other for AP2 would greatly enhance our understanding of how adaptors and cargo are spatially and temporally regulated *in vivo*.

The clathrin TD has been identified as the major interaction site on clathrin for adaptor proteins. Initial crystallographic structures identified 3 potential binding sites for specific adaptor motifs (Figure 5B), suggesting that adaptors would bind to discrete locations on the TD (ter Haar et al., 2000; Miele et al., 2004; Kang et al., 2009). However, recent studies both *in vivo* and *in vitro* have identified a 4^th^ binding site (Figures 5B,F) and present evidence that the binding of these motifs is much more degenerate than previously expected, with peptides of a given sequence found to bind in more than one location (Willox and Royle, 2012; Zhuo et al., 2015; Muenzner et al., 2017). As with AP2-adaptor interactions, a greater understanding of the relative affinities of these motifs or associated proteins would give us better insight into how adaptor recruitment is regulated in CME.

To conclude, clathrin-mediated endocytosis is a versatile pathway, not just in terms of the diversity of cargos that can be internalized, or in the large number of accessory and adaptor proteins used, but it also in the pivotal role of weak molecular interactions orchestrating and controlling the internalization of specific cargo and its delivery to early endosomes.

## Author contributions

All authors (SS, MB, MH, and CS) fulfill the “author criteria” of: Substantial contributions to the conception or design of the work; or the acquisition, analysis, or interpretation of data for the work; and Drafting the work or revising it critically for important intellectual content; and Final approval of the version to be published; and Agreement to be accountable for all aspects of the work in ensuring that questions related to the accuracy or integrity of any part of the work are appropriately investigated and resolved.

### Conflict of interest statement

The authors declare that the research was conducted in the absence of any commercial or financial relationships that could be construed as a potential conflict of interest.
